# An Asymmetric Wall-Thickening Pattern Predicts Response to Cardiac Resynchronization Therapy

**DOI:** 10.1016/j.jcmg.2018.01.022

**Published:** 2018-10

**Authors:** David R. Warriner, Tom Jackson, Ernesto Zacur, Eva Sammut, Paul Sheridan, David Rod Hose, Patricia Lawford, Reza Razavi, Steve Alexander Niederer, Christopher Aldo Rinaldi, Pablo Lamata

Cardiac morphology changes in heart failure and provides information on the state of the heart. We hypothesized that pre-implant left ventricular (LV) morphology differs significantly between responders and nonresponders to cardiac resynchronization therapy (CRT). To this end, the LV morphology of 50 subjects selected for CRT was studied from cardiac magnetic resonance by building a statistical 3-dimensional (3D) anatomic atlas.

Fifty consecutive subjects—39 men, 69.3 ± 11.2 years of age, 46% with ischemic etiology, and 92% with left bundle branch block (LBBB)—underwent a cardiac magnetic resonance anatomic study: electrocardiogram-gated free-breathing steady-state free-precession 3D anatomic sequences with nearly isotropic spatial resolution (median of 0.89 × 0.89 × 1.00 mm) before the CRT implantation procedure with a lead in a lateral or posterolateral vein as per standard clinical practice. They were all clinically assessed at baseline and followed up 6 months after implantation: LV end-systolic volume change on echocardiography defined CRT response, positive with a reduction in LV end-systolic volume of >15% (1). Two patients withdrew; 25 patients were identified as responders and 23 as nonresponders. This study was approved by the local National Health Service (NHS) health research authority (NRES number 10/H0802/71).

A statistical anatomic atlas was built from the 50 steady-state free-precession datasets involving: 1) a semiautomatic segmentation of the LV by 2 observers, reaching a Dice similarity metric with fully manual approach of 0.74 ± 0.05; 2) the automatic construction of 3D models (2), reporting an average fitting error of 0.45 mm; 3) generation of the consensus in each case (i.e., the average model) between the 2 observers; and 4) the generation of the statistical model by a principal component analysis of the 50 shapes after subtraction of the center of mass, requiring 19 modes to reconstruct shapes with a median error <0.5 mm (one-third of voxel size).

**Figure 1:**
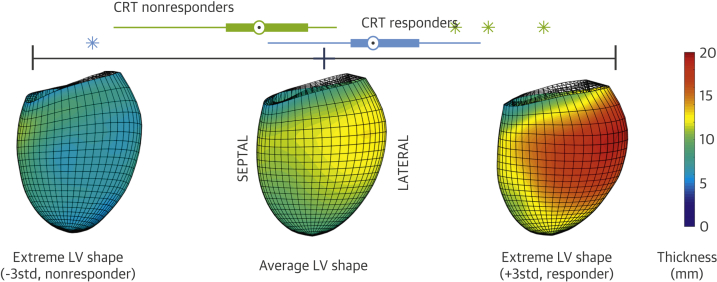
3D Remodeling Signature Predictive of CRT Response Remodeling signature predictive of cardiac resynchronization therapy (CRT) response, illustrating the box-plot distribution of responders and nonresponders, and the 3-dimensional (3D) left ventricular (LV) anatomy (mean and both extremes of the distribution) color-coded accordingly to the thickness of the myocardial wall.

LV shape changes due to a variety of factors, and the goal was to identify the LV morphological pattern that is most associated with CRT response. We thus found the linear combination of principal component analysis modes that best differentiated response to CRT through a linear discriminant analysis. Linear discriminant analysis performance was evaluated by cross-validation (1,000 repetitions of a leave-4-out test), identifying the combination that maximized the area under the curve of the receiver-operating curve, finding an area under the curve of 0.7603 ± 0.0162, and a sensitivity and specificity of 77% and 82%, respectively, at the optimal working point. The visual inspection of the remodeling signature that was predictive of CRT response (Figure 1) revealed an angulation of the basal plane in the septolateral direction (relative shorter septal wall and longer lateral wall in the nonresponder) and an irregular thickening pattern, whereas responders showed thicker walls in the lateral and basal regions compared with nonresponders. Results do not suggest to capture the extreme wall thinning of non-viable ischemic regions (see −3std thickness map in Figure 1).

The presence of a thicker lateral wall in the responder suggests a larger workload localized in this region causing the localized thickening. This is supported by earlier studies using positron emission tomography, which demonstrated metabolism was highest in the lateral wall and lowest in the septum at baseline in all patients (3). The specific localization in the lateral wall is also consistent with in silico results that showed the imbalance of work distribution in the presence of a LBBB, where the lateral wall is the one that produces the largest work rate (4). We then speculate that, in the presence of LBBB, the lack of this local remodeling response (i.e., the LV wall getting thinner and not thicker) reflects the lack of the myocardial tissue to cope with the extra work burden. And this impaired adaptation is a signature that predicts the impaired capability of the heart to positively respond to the resynchronization procedure.

In conclusion, a specific morphological signature, with asymmetric thickness in the pre-implant LV shape, was found to be an independent predictor of a favorable remodeling response to CRT. LV shape may represent a potential new criterion for CRT patient selection.
